# Cancer-Associated Fibroblasts as Another Polarized Cell Type of the Tumor Microenvironment

**DOI:** 10.3389/fonc.2014.00062

**Published:** 2014-03-27

**Authors:** Martin Augsten

**Affiliations:** ^1^Department of Oncology-Pathology, Cancer Center Karolinska, Karolinska Institutet, Stockholm, Sweden

**Keywords:** cancer, tumor microenvironment, cancer-associated fibroblasts, tumor promotion, tumor suppression, polarization

## Abstract

Tumor- or cancer-associated fibroblasts (CAFs) are one of the most abundant stromal cell types in different carcinomas and comprise a heterogeneous cell population. Classically, CAFs are assigned with pro-tumorigenic effects stimulating tumor growth and progression. More recent studies demonstrated also tumor-inhibitory effects of CAFs suggesting that tumor-residing fibroblasts exhibit a similar degree of plasticity as other stromal cell types. Reciprocal interactions with the tumor milieu and different sources of origin are emerging as two important factors underlying CAF heterogeneity. This review highlights recent advances in our understanding of CAF biology and proposes to expand the term of cellular “polarization,” previously introduced to describe different activation states of various immune cells, onto CAFs to reflect their phenotypic diversity.

## Introduction

Tumors display an organ-like structure and are composed of reciprocally interacting cell types including cancer-initiating cells, more or less differentiated cancer cells, extracellular matrix, and a variety of stromal cells such as endothelial cells, immune cells, pericytes, adipocytes, and fibroblasts. Tumors produce a multitude of different factors such as growth factors, cytokines, and chemokines that affect the phenotype and function of tumor-resident cells and impact on the composition and texture of the extracellular matrix, thereby modulating, e.g., tumor stiffness. Among the tumor-derived, secreted factors are various signaling mediators that promote tumor malignancy through local and systemic signals, i.e., these factors can affect tumor-distant tissues and organs. Some of those signals are known to act on the bone-marrow and stimulate the release of bone-marrow derived cells (BMCs) that, e.g., prepare the pre-metastatic niche for seeding cancer cells and support their survival and outgrowth in the new soil (1, 2). The various cell types of a tumor exhibit an enormous grade of plasticity when exposed to the cocktail of tumor-derived factors. For example, cancer cells acquire novel properties ensuring their survival, enabling their expansion, and enhancing their malignant behavior (3). Depending on the type of stimuli, cells of the tumor microenvironment can adopt different activation states that are associated with phenotypes ranging from tumor promotion to tumor suppression (4).

Cancer-associated fibroblasts (CAFs), originally introduced as carcinoma-associated fibroblasts (5), represent one of the most abundant stromal cell types of several carcinomas including breast and prostate cancer. CAFs are activated fibroblasts that share similarities with fibroblasts stimulated by inflammatory conditions or activated during wound healing (6, 7). Different cellular origins and tumor-derived factors shape the phenotype of CAFs and contribute to their appearance as heterogeneous cell population with distinct subtypes. Besides their established role as promoter of tumor growth and progression, recent data obtained from *in vitro* co-cultures and *in vivo* xenograft models, suggest a tumor-inhibitory role of CAFs. This review will discuss the different elements that, taken together, contribute to the plasticity and diverse phenotypes of CAFs.

## Cellular Marker Define Cancer-Associated Fibroblasts as a Heterogeneous Cell Population

Comparative gene expression profiling of tissue-derived normal fibroblasts (NFs) and CAFs and other approaches revealed that CAFs produce a variety of factors which are lower or not expressed by the normal counterparts. As a sign of their activation, CAFs produce several mesenchyme-specific proteins such as fibroblast-specific protein (FSP-1), also known as S100A4, the fibroblast-activating protein (FAP), vimentin, and alpha-smooth muscle actin (α-SMA), the prototypical marker for myofibroblasts. CAFs are also a rich source of different secreted factors such as cytokines, chemokines (e.g., IL-6, CXCL8, CXCL12), and growth factors including vascular endothelial-derived growth factor (VEGF), transforming growth factor beta (TGF-β), hepatocyte growth factor (HGF), epidermal growth factor (EGF), or fibroblast growth factor (FGF), and express receptors such as platelet-derived growth factor receptor alpha (PDGFRα) and platelet-derived growth factor receptor beta (PDGFRβ). These soluble factors are involved in paracrine signaling or activate CAFs in autocrine loops, thereby contributing to the constitution of the CAF phenotype. Furthermore, CAFs play an important role in remodeling of the extracellular matrix by expressing a wide variety of matrix-components and matrix-remodeling enzymes such as neuron glial antigen (NG2), tenascin C, type I collagen, fibronectin, or MMP-1/stromelysin-1 (8, 9).

Several intracellular and plasma membrane-associated proteins such as α-SMA, vimentin, and PDGFRβ have been used as CAF markers to detect CAFs in tumor tissue. These markers were also used in functional studies to purify CAFs from tissue or to study their role in tumor growth and metastasis (10–14). A recent study by Orr et al., revealed additional markers such as ASPN, ZEB1, and OGN that distinguish prostate CAFs from their normal counterparts (15). The glycoprotein podoplanin was also suggested as another, novel CAF-marker that has been shown to have prognostic significance for different types of tumors (16, 17). However, all the CAF markers described so far are apparently not unique for this cell type and are expressed by other cell types of a tumor, reflecting the plasticity of stromal cells. For example, podoplanin is also a marker of lymphatic vessels and can be expressed by cancer cells, and expression of the PDGFRβ is also a feature of pericytes (16, 18). This makes it difficult to purify CAFs on basis of markers from other cells like adipocytes, endothelial cells, or pericytes that share expression of those proteins. Thus, many cancer studies claiming to describe CAF-specific effects, and relying on the use of shared markers, might be flawed by inclusion of other cell types. If there is not a single CAF-specific marker perhaps a combination of different markers will eventually help to better define the CAF population in the future.

Fibroblasts comprise a heterogeneous population of mesenchymal cells. NFs exhibit a “topographic differentiation” pattern, i.e., fibroblasts express a transcriptional program associated with their anatomical location (19). Accordingly, topographic differentiation is likely to affect the appearance of CAFs and contributes to (systematic) differences among CAFs from different anatomical sites. But also within the same type of tissue, CAF markers are not uniformly expressed on these cells and rather define distinct CAF subsets. The study by Sugimoto et al. was the first to describe different CAF-subtypes based on expression analysis of FSP-1/S100A4, PDGFRβ, NG2 α-SMA in a pancreatic and a breast cancer mouse model (9). Interestingly, the findings from both tumor models were remarkably similar and revealed one CAF-subtype that is characterized by co-expression of α-SMA, PDGFRβ, and NG2, while FSP-1 expression defines another CAF-subtype. Furthermore, gene expression analysis demonstrated a breast cancer subtype-specific molecular profile of CAFs (20). Despite these efforts, it is still not known how many CAF-subtypes exist in a certain tumor type and how the tentative CAF subsets are associated with different tumor compartments. The question if different, marker-defined CAF subsets exert different functions is emerging and has been started to address (14, 21–24).

## To the Roots of Cancer-Associated Fibroblasts

Different elements contribute to the heterogeneity of CAFs including the tissue type in which the tumor grows, the local paracrine environment, and the cell type-of-origin. One significant source for CAFs is local fibroblasts and tissue-resident fibroblast precursor cells that become incorporated into the growing tumor by tumor-derived stimulants (25).

As introduced above, tumors produce and release a variety of factors such as chemokines with long-ranging effects that modulate the phenotype of cells in a tumor-distant environment and eventually attract cells from a distant site into the tumor. Here, the bone-marrow attracted much attention as a site that is subjected to extensive changes by tumor-derived systemic signals (2, 26). Tumor-activated BMCs are implicated in the formation of the pre-metastatic niche and can also differentiate into CAFs upon recruitment into tumors (27–29). Furthermore, once entered the tumor BMCs have been shown to attract local fibroblast that adopt a CAF phenotype in the tumor milieu (25). Among the BMCs, mesenchymal stem cells (MSCs) attracted much attention because they can differentiate in a variety of different stromal cells like CAFs depending on the factors and structures they are exposed to (8, 30). In the tumor microenvironment, MSCs not only differentiate into CAFs but can, for example, also give rise to endothelial and various immune cells (31).

The conversion of differentiated cells represents another source of CAFs and provides an impressive example for the enormous plasticity that cells exhibit when exposed to the environment of a tumor. Epithelial-to-mesenchymal transition (EMT), one of the developmental processes hijacked by tumors, contributes to an invasive, pro-metastatic phenotype of cancer cells and gives rise to mesenchymal-like cells that share marker and properties with CAFs (32). Interestingly, CAF-derived signals in turn can also promote EMT and contribute to cancer stemness (33–35). In an EMT-related process termed endothelial-to-mesenchymal transition (EndMT), tumor-derived signals stimulate trans-differentiation of endothelial cells to adopt a CAF-like phenotype, characterized by expression of fibroblast marker (α-SMA, FSP-1) and the endothelial marker CD31 (36). Although not formally established, vessel-associated, α-SMA-expressing pericytes have been suggested as an additional origin for CAFs (18). Furthermore, adipocytes can be subjected to the instructive force of the tumor milieu and also contribute to the CAF population (37).

The different sources represent an important component that contributes to the heterogeneity of CAFs and contributes to the difficulty to distinguish CAFs from other cell types expressing similar markers. However, little is known if different tumor types share certain sources for CAFs, if the different cellular origins give rise to specific CAF-subtypes and to what extent the different sources contribute to the whole of the CAF population of a given tumor. A recent study analyzing the fraction of α-SMA-positive myofibroblasts in kidney fibrosis determined that approximately 65% of myofibroblasts in inflamed tissue are locally resident fibroblast that become activated under these conditions. The remaining 35% of myofibroblasts are derived from BMCs recruited into the kidney by pro-inflammatory signals (38). In the context of cancer, Quante et al. found that approximately 20% of the myofibroblasts in an inflammation-induced gastric cancer model were bone-marrow derived (29). In a pancreatic cancer model, BMCs contributed with up to 40% to the α-SMA-positive CAF population (28). However, in a breast cancer model studying systemic signaling, myofibroblasts in tumors growing at a distant site were derived from local sources but not from BMCs (25), indicating tissue type or cancer model-specific differences. Furthermore, EndMT can account for up to 12% of α-SMA-positive and 30% of FSP-1-positive CAFs in a murine melanoma model (36). And Kidd et al. found that approximately 29% of α-SMA-positive fibroblasts were derived from breast adipose tissue (39). The latter study also suggested that the relative proportion of different CAF-subtypes varies in a tumor (39).

## Tumor-Promoting Effects of Cancer-Associated Fibroblasts

Intensive research over the last decade revealed that CAFs play a pivotal role in the multi-step process of tumorigenesis, pro-metastatic signaling, and metastatic growth, impacting on each step. Although still under debate if CAFs by themselves can induce malignant transformation of “normal” epithelium (lacking oncogenic mutations), there is compelling evidence that CAFs promote tumor development and progression from pre-malignant stages (5, 14), stimulate metastasis (40, 41), and support the outgrowth of disseminated cancer cells at the site of metastasis (42). It has also been recognized that different CAF subsets, such as those characterized by α-SMA or FSP-1, display a pro-tumorigenic phenotype (14, 43). However, it is less clear if fibroblast subpopulations stimulate distinct aspects during malignancy.

**Figure 1:**
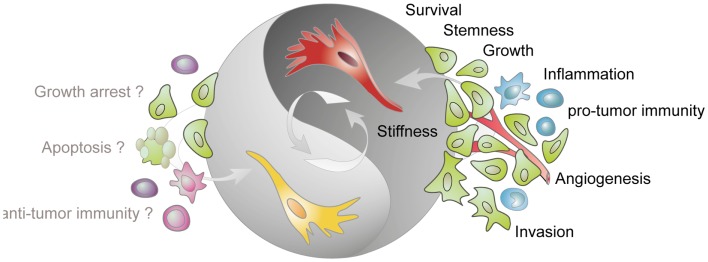
**Tumor-stimulatory and tumor-inhibitory effects of CAFs**. Fibroblasts present in the tumor stroma (CAFs) are predominantly assigned with a tumor-promoting function. CAFs (shown in red) stimulate cancer cell survival, growth, and invasion, enhance the stiffness of the extracellular matrix, contribute to angiogenesis by releasing pro-angiogenic factors, contribute to a pro-inflammatory milieu, and impact on the activation state of various immune cells. More recent data demonstrate that tumor-resident fibroblast (depicted in yellow) can also confer tumor-suppressive effects. However, the mechanisms underlying this inhibitory phenotype are not known but may involve direct inhibition of cancer cells and modulation of immune cell behavior.

The pro-tumorigenic activity of CAFs includes strong paracrine effects impacting on different cell types present in the tumor (Figure 1). Direct stimulation of cancer cells by CAF-derived signals promotes, e.g., cancer cell proliferation (44, 45), migration, invasion (46–50), and the adoption of a cancer stem cell phenotype by inducing EMT (51–55). Interestingly, CAFs have also been shown to pave the way for cancer cells, providing tracks for cancer cells to migrate and invade surrounding tissue (56, 57). Furthermore, CAFs secrete a variety of pro-inflammatory factors (21, 58) leading to the recruitment and promotion of immunosuppressive (59) and tumor-promoting immune cells (60), thereby contributing to the establishment of a pro-inflammatory, immune-suppressive, tumor-permissive environment. The various pro-tumoral activities of CAFs are comprehensively covered by a number of recent reviews that are recommended for further reading (61–64).

Along with these studies an ever-expanding list of CAF-derived, pro-tumorigenic factors (e.g., growth factors, cytokines, chemokines, matrix-components, and matrix-remodeling enzymes) and associated signaling pathways (e.g., TGF-β, Wnt, Hedgehog) have been identified. Many of these factors stimulate several CAF-activities and affect multiple cell types. For example, the chemokine CXCL12 has been shown to promote tumor growth by affecting breast cancer cell growth and by stimulating the recruitment of endothelial precursor cells (EPCs) into the tumor (65). Some CAF-derived factors such as the chemokine CXCL14 exert autocrine effects on fibroblasts (66), while other CAF-derived factors act in a paracrine manner stimulating cancer and stromal cells (e.g., endothelial cells, immune cells), and are involved in the recruitment of host cells into the tumor (29, 40, 67). Moreover, CAF-derived matrix-components and matrix-remodeling enzymes can, e.g., increase the stiffness of solid tumors thereby enhancing aggressiveness and stimulating metastasis (68–70).

## Tumor-Suppressive Effects of Tumor-Resident Fibroblasts

Tumor promotion is the dominating functional property allocated to CAFs, and together with enhanced proliferation used as a marker to discriminate CAFs from NFs (71). In contrast, NFs have the capacity to suppress growth and progression of pre-malignant lesions (72).

Recent data suggest that this ability can also be a feature of tumor-resident fibroblasts (Figure 1). For example, primary fibroblasts established from normal and cancer tissue can inhibit the growth of a panel of co-cultured cancer cells *in vitro* (73). More mechanistic insight was recently provided by Chang et al. using primary fibroblasts established from breast tissue (74). Both, NFs and CAFs expressing the ligand Slit2 inhibited the tumorigenicity of breast cancer cells expressing the corresponding Robo1-receptor on their surface. Ligand-induced Robo1 activation interfered with PI3K- and β-catenin signaling in cancer cells and diminished their malignant potential. Previously, it was shown that Slit-stimulated signaling also inhibits the pro-tumorigenic SDF1/CXCR4-signaling pathway (75, 76). In contrast, the activity of breast cancer cells lacking Robo1-expression was rather stimulated by Slit2-expressing fibroblasts, demonstrating that the functional outcome of CAF activity is decided on the level of the malignant cells (74). Importantly, tissue analyses revealed the presence of Slit2-expressing fibroblasts in breast tumor tissue thus validating the relevance of these CAFs in human disease and suggesting Slit2 as a potential effector of this tumor-inhibitory CAF-subtype. Furthermore, Slit expression was demonstrated to have prognostic significance predicting overall survival and occurrence of metastasis (74).

In another study, Green et al. observed that fibroblast-derived Wnt3a could promote but also inhibit the growth of different, orthotopically growing patient-derived breast xenograft tumors (77). However, the molecular basis for the opposing behavior of Wnt3a-expressing fibroblasts remained unresolved. Nevertheless, these recent studies provide first clear experimental evidence that the same type of CAF can exert a broader spectrum of activities ranging from tumor stimulation to tumor inhibition. Moreover, the impact of CAFs on tumorigenesis appears to be less dependent on the instructive role of CAFs but is rather governed by the interacting malignant compartment. This illustrates the strong context-dependent action of CAFs in the tumor milieu as previously described for other stromal cells such as immune cells and pericytes (4, 18).

The wide range of fibroblast activities is defined by their molecular makeup that is controlled by an intrinsic expression program related to the fibroblast site of origin, and modulated by external cues. Accordingly, CAFs express a variety of different factors that contribute to shape the environment, including pro-tumorigenic factors that potently stimulate tumor growth. However, the same CAFs may eventually co-express factors that by themselves suppress the action of tumor-resident cells. For example, primary prostate CAFs express several tumor-promoting factors but at the same time molecules have been shown to suppress cancer cell growth, migration, and invasion (Martin Augsten, unpublished observation). Furthermore, individual CAF-derived factors also act in a cell type and/or tumor stage-dependent manner. For example, the chemokine CXCL14 is expressed by cancer cells and CAFs of different tumor types, and CXCL14 acting through fibroblasts exerts tumor-promoting effects *in vivo* by stimulating angiogenesis and macrophage infiltration (66, 78). In contrast, CXCL14 expressed by cancer cells inhibits the growth of xenograft tumors derived from different origins (79, 80). Similarly, Wnt signaling has been shown to critically contribute to tumorigenesis (42, 81). On the contrary, the fibroblast-derived Wnt-ligand Wnt3a can promote and inhibit breast cancer tumor growth by yet unknown mechanisms (77). Furthermore, TGF-β, for which CAFs are an important source, is known to suppress tumor initiation and early tumor growth but promotes tumor progression and metastasis (82, 83). As discussed above, Slit2-induced Robo1 signaling was identified as one mechanism by which CAFs exert a tumor-suppressive effect (74). Thus, it will be interesting to elucidate the molecular mechanisms underlying the differential action of other CAF-derived factors such as Wnt3a, CXCL14, and GDF15, a divergent member of the TGF-β superfamily. Further studies should aim to understand the relative contribution of inhibitory and stimulatory signals to the CAF phenotype and analyze the expression pattern and kinetics of these signals during the course of disease.

Although the potential tumor-inhibiting effect of CAFs is by far less studied as their tumor-promoting activity, the data available imply that CAFs exhibit a much broader spectrum of activities than previously demonstrated. CAFs and CAF-derived factors strongly act in a context-dependent fashion, and their tumor-promoting and/or tumor-inhibiting activity is determined by the intrinsic properties of CAFs but also depends – perhaps to an even larger extent – on how these signals are processed by the tumor environment, i.e., the type and/or the state of malignant and other stromal cell populations. In that sense, CAFs retain features of “normal” fibroblasts (fibroblasts in a tumor-free host) that act as sensors of their environment and await activation by external cues such as TGF-β, PDGF, IL-1. Of note, this is not to confuse fibroblasts for an exclusively passively acting cell type (that solely integrates external signals) since fibroblasts including CAFs instruct their environment (normal and malignant epithelium) upon stimulation (5, 72).

## Assigning the Polarization Concept to CAFs

The plasticity and emerging functional divergence of CAFs challenges our current definition of CAFs as a tumor-resident stromal cell type assigned with tumor-promoting activity. Other cells of the tumor microenvironment such as immune cells display also an enormous grade of plasticity, and the term “polarization” has been introduced to describe different activation states that immune cells adopt in response to different external cues (4). Type I and type II mark the two ends of the polarization spectrum, and represent distinct cellular lineages associated with different markers and opposing activities in tumors. In the complex, heterogenic (micro)milieu of a tumor immune cells apparently move in a continuum of activation states between type I and type II.

Macrophages provide an illustrative example and can be polarized into type I (“M1”) and type II (“M2”) macrophages, respectively. While M1-polarized macrophages exert tumor-suppressive effects, M2-polarized macrophages, called tumor-associated macrophages (TAMs), promote tumor growth and progression (84, 85). Importantly, the polarization of macrophages can be controlled by specific factors that are derived from autocrine and/or paracrine signaling. For example, treatment of macrophages with TNF-α, IFN-γ, and LPS *in vitro* induces type I polarization, while macrophages adopt a type II phenotype under the influence of IL-4, IL-10, and IL-13 (86). Polarization appears to be a more general phenomenon because also various other immune cells such as T-cells, dendritic cells, and neutrophils can adopt different activation states under the influence of a tumor (87–89). Interestingly, the different tumor-associated cell types actively regulate the polarization status of each other. For example, CAF-derived signals can promote polarization of CD4^+^ T-cells to adopt a tumor-supportive, Th2 phenotype that is associated with enhanced infiltration of TAMs and regulatory T (Treg) cells (90). Furthermore, CAFs secrete IL-6 and CCL2 thereby promoting the development of M2-macrophages (21, 60).

As introduced in the previous chapters, the phenotype of tumor-associated fibroblasts is shaped by two components: the type of cell giving rise to CAFs and the local environment CAFs are embedded in. Exposure of fibroblasts to growth factors, cytokines, reactive oxygen species (ROS), or a stiff matrix can induce a CAF phenotype characterized by, e.g., α-SMA expression and endowed with the ability to promote tumor growth and progression (33, 68). On the contrary, the pro-tumorigenic action of CAFs can be reverted by exposure to certain tumor-inhibitory factors such as TGFBI (43), and tumor-resident fibroblasts can suppress tumor growth in a proper microenvironment (74, 77). These recent developments in the field extend our current view on CAFs and provide *in vivo* evidence of a similar degree of plasticity of tumor fibroblasts as previously shown for different tumor-associated immune cells.

Thus, I would like to propose to take on the concept of “polarization” used in the context of immune cells and extend this concept onto CAFs. Hence, the term “CAFs” defines a population of fibroblasts present within the tumor or associated with the tumor margin but does not, in contrast to the original definition, assign a particular function to these cells. It seems to be about time to revisit our definition on CAFs to adopt that to the increasing knowledge around this cell type. Li et al. discussed the “two-faced characteristics of fibroblasts in tumor stroma” in a recent review (61). In an effort to better reflect the dynamic state of tumor-associated fibroblasts, Mader et al. introduced the term “CAF state” to describe the *marker*-based heterogeneity of these cells (91). The herein proposed concept of “CAF polarization” highlights the *functional* heterogeneity of CAFs and proposes that the different activation states of CAFs are associated with different, as yet poorly described marker. Thus, both definitions are not mutual exclusive but rather aim to provide a starting point to interconnect CAF-marker with specific functional phenotypes.

**Figure 2:**
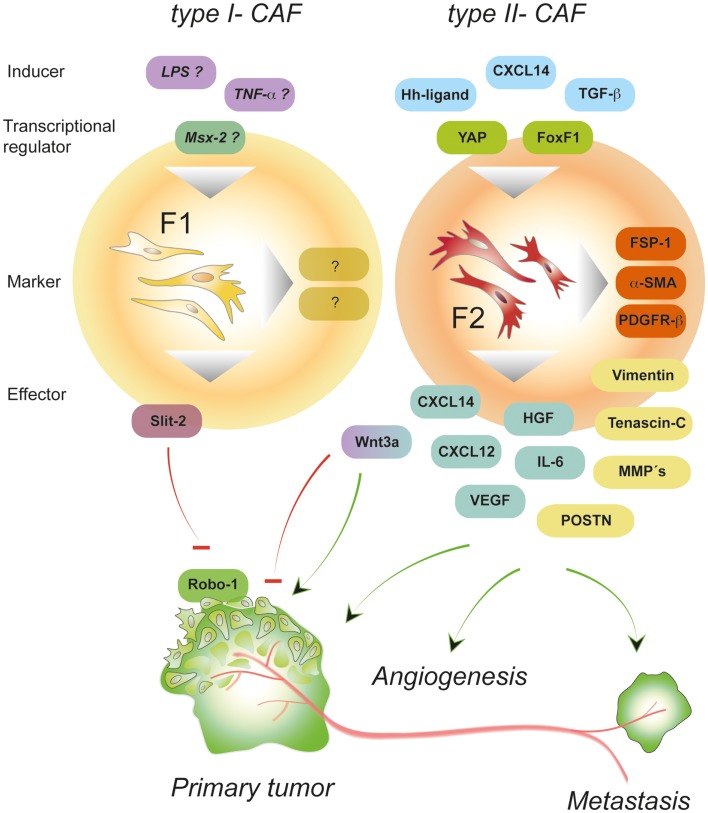
**Properties of polarized CAFs**. Depending on the type of signals, intra-tumoral fibroblasts can be polarized thereby adopting different activation states. Type II polarized fibroblasts (F2) differentiate into pro-tumorigenic fibroblasts under the influence of, e.g., growth factors and chemokines (called “inducer”). Signaling induced by CXCL14, hedgehog (Hh), or TGF-β activates transcription factors that induce a program controlling the expression of a variety of “effector”-molecules (matrix-components, matrix-remodeling enzymes, growth factors, and cytokines) that in turn deploy pro-tumorigenic effects by stimulating other cells in the environment of the local tumor and beyond. In contrast, type I polarized fibroblasts (F1)-expressing molecules such as Wnt3a and Slit2 can exhibit tumor-inhibitory effects by suppressing the action of cancer cells. About the exogenous signals and transcription factors involved in establishing the tumor-suppressive phenotype can only be speculated so far. Potential inducers of type I-CAFs are TNF-α and LPS that have been shown to stimulate Wnt3a and chemokine expression, respectively (92).

Adopting the style introduced for the different polarized immune cells, CAFs will be divided into two functionally different subtypes: F1- and F2-polarized fibroblasts (Figure 2). F1 represents type I-CAFs displaying tumor-inhibitory effects while F2 refers to type II-CAFs that exhibit tumor-promoting properties. The obvious heterogeneity of CAFs and the recent findings on Slit2- and Wnt3a-expressing, tumor-suppressive fibroblasts (74, 77) suggest that: (1) F1 and F2 represent the two extremes of a spectrum of different phenotypes that CAFs can adopt and (2) the polarization of CAFs stays under the control of the tumor (micro)environment. In contrast to polarized immune cells where several marker associated with either the type I or the type II subtype have been described, the marker/function relationship for CAFs is much less characterized. Thus, the molecular makeup of polarized fibroblasts, and the F1-type in particular, remain largely elusive (Figure 2). Because the action of CAFs depends on the nature of the interacting cell type it appears as an important future task to identify marker associated with distinct CAF activation states. Further studies should also aim to address if these activation states are associated with a specific tumor stage.

## Concluding Remarks

Much progress has been made in our understanding of CAFs and revealed their multifaceted contributions to tumor development, progression, and metastasis. A tumor-inhibitory action of this cell type is also emerging but needs further validation. The findings together imply that CAFs confer a much broader range of action than previously thought and exhibit a similar degree of plasticity as described for other cells of the tumor microenvironment. Expanding the concept of cellular polarization to CAFs, as proposed here, will accommodate for the functional diversity of CAFs and aims to provide a framework to delineate unresolved questions around the different CAF phenotypes. For example, it is not clear which factors induce and determine certain CAF-subtypes. An important task that has begun to be addressed is to catalog the different CAF-subtypes present within a given tumor type and across different types of tumors. However, it is unclear how many CAF populations exist, and which marker are associated with the tumor-promoting and tumor-inhibiting phenotype, respectively. Addressing these questions will help to reveal if populations of tumor-inhibitory CAFs are always present in the tumor microenvironment, and if this population can be activated or strengthened to limit initial and progressive tumor growth. Of note, the recent data implicate that the tumor environment defines the polarization status of CAFs that will adopt a tumor-permissive, tumor-promoting, or tumor-inhibitory phenotype depending on the stimuli. It is likely, yet to be substantiated, that certain tumor-derived factors alone or in a combination will shift the CAF polarization status, in extreme scenarios from type I to type II or vice versa. Considering the heterogeneity of a tumor (93), a certain CAF-subtype might exhibit suppressive effects in a certain microenvironment of the tumor while acting stimulatory in another. Thus, a deeper and more profound understanding of CAF biology is required if CAFs will be successfully explored as therapeutic targets.

## Conflict of Interest Statement

The author declares that the research was conducted in the absence of any commercial or financial relationships that could be construed as a potential conflict of interest.
